# A Narrative Review of Recent Advances in Rapid Assessment of Anthocyanins in Agricultural and Food Products

**DOI:** 10.3389/fnut.2022.901342

**Published:** 2022-07-19

**Authors:** Muhammad Faisal Manzoor, Abid Hussain, Nenad Naumovski, Muhammad Modassar Ali Nawaz Ranjha, Nazir Ahmad, Emad Karrar, Bin Xu, Salam A. Ibrahim

**Affiliations:** ^1^School of Food and Biological Engineering, Jiangsu University, Zhenjiang, China; ^2^Department of Agriculture and Food Technology, Faculty of Life Science, Karakoram International University, Gilgit-Baltistan, Pakistan; ^3^School of Rehabilitation and Exercise Science, Faculty of Health, University of Canberra, Canberra, ACT, Australia; ^4^Functional Foods and Nutrition Research (FFNR) Laboratory, University of Canberra, Bruce, ACT, Australia; ^5^Institute of Food Science and Nutrition, University of Sargodha, Sargodha, Pakistan; ^6^Department of Nutritional Sciences, Faculty of Medical Sciences, Government College University Faisalabad, Faisalabad, Pakistan; ^7^State Key Laboratory of Food Science and Technology, School of Food Science and Technology, Jiangnan University, Wuxi, China; ^8^Food Microbiology and Biotechnology Laboratory, North Carolina Agricultural and Technical State University, Greensboro, NC, United States

**Keywords:** anthocyanin, chemometrics, non-destructive techniques, agricultural product, food products

## Abstract

Anthocyanins (ACNs) are plant polyphenols that have received increased attention recently mainly due to their potential health benefits and applications as functional food ingredients. This has also created an interest in the development and validation of several non-destructive techniques of ACN assessments in several food samples. Non-destructive and conventional techniques play an important role in the assessment of ACNs in agricultural and food products. Although conventional methods appear to be more accurate and specific in their analysis, they are also associated with higher costs, the destruction of samples, time-consuming, and require specialized laboratory equipment. In this review article, we present the latest findings relating to the use of several spectroscopic techniques (fluorescence, Raman, Nuclear magnetic resonance spectroscopy, Fourier-transform infrared spectroscopy, and near-infrared spectroscopy), hyperspectral imaging, chemometric-based machine learning, and artificial intelligence applications for assessing the ACN content in agricultural and food products. Furthermore, we also propose technical and future advancements of the established techniques with the need for further developments and technique amalgamations.

## Introduction

Anthocyanins (ACNs) are water-soluble phenolic compounds that are identified as pigments (red, purple, and blue) of various plants and are traditionally used as natural food colorants. They are also regarded as bioactive compounds with high antioxidant properties when consumed individually or as part of a healthy diet (1–3). The richest sources of ACNs are identified in several different berries (strawberries, blackcurrants, blackberries, blueberries, redcurrants, raspberries, and cranberries), with levels ranging from 100 to 700 mg/100 g of fresh weight. Nevertheless, the highest level of ACNs is found in elderberries and chokeberries (4, 5). Furthermore, ACNs are localized in different sections of the fruits such as skin and flesh and, to date, there are over 700 structurally varied ACNs that have been identified. This number is expected to increase in the future with improvements in technological advancements and the identification of different sources of ACNs (6).

The ACNs are the glycoside form of anthocyanidins, the water-soluble form of vacuolar pigments, which has a sugar-free form. The color of both groups of compounds is dependent on environmental acidity. Anthocyanidins are known as aglycones and are grouped into *O*-methylated anthocyanidins, 3-deoxyanthocyanidins, and 3-hydroxyanthocyanidins. The most common anthocyanidins include pelargonidin, malvidin, peonidin, petunidin, delphinidin, and cyanidin (7). Acylated ACNs are also found in plants besides the “typical” ACNs and are further classified into coumaroylated ACNs, caffeoylated ACNs, acrylated ACNs, and malonylated ACNs.

The consumption of ACNs has been associated with several beneficial health effects such as a reduced risk of developing cardiovascular disease (CVD), improvements in cognition, and a reduction in inflammation (5). These effects are proposed to be due to the ACNs strong scavenging activity against reactive oxygen and nitrogen-free radical species (5, 8, 9). Relatively recent studies have identified that the consumption of different food products rich in ACNs can potentially reduce the CVD risk factors *via* the improvements in endothelial function and reduction in inflammatory responses that are associated with the consumption of typical Western dietary patterns (10). Inflammatory markers in older adults that live with mild cognitive impairment were reduced by the consumption of ACN-rich fruit juice (11), and the consumption of fruits (blueberries) high in ACNs exhibited beneficial cognitive outcomes with improvements in short- and long-term memory and spatial memory (12). These beneficial health outcomes were proposed to be associated with the improvements in microbial diversity of the gut-microbiota (13). The findings from observational studies indicate that regular consumption of red wine (rich in ACNs and polyphenols) seems to improve α-diversity, in particular, *Barnesiella spp* which may be associated with improved cholesterol metabolism and body composition (14).

With the global increase in demand for high-quality and nutrient-dense foods (15), there is a strong focus on non-invasive and rapid methods of detection with high sensitivity and accuracy for several different phytochemicals (16–18). Recently, several reviews have discussed the application of non-destructive spectroscopic and spectral imaging techniques for various agricultural applications such as exploring the maturity and ripening of the fruits, lycopene content, quality assessment of cheeses, potatoes, and detecting insect infestation in fruits and vegetables (19–24). However, to the best of our knowledge, there are no reviews that summarize the most recent evidence on the use of rapid and non-destructive techniques for the assessment of ACN contents in agricultural products. Therefore, the main aim of this literature review is to present a summary of the rapid and non-interruptive methods and technologies for the evaluation of ACN in different agricultural products and food items.

## Data Screening and Methodology

In May 2021, non-systematic searches were performed in five electronic databases (Science Direct, Google Scholar, SCOPUS, and PubMed) using the following search strategy: “anthocyanins” AND “non-destructive techniques,” “Raman spectroscopy,” “hyperspectral imaging,” “surface-enhanced Raman spectroscopy.” Only articles published in English were included in this review. A large number of articles were focused on the chemistry, health benefits, and extraction techniques of several different ACNs. Included articles were further grouped based on the overall non-destructive techniques implemented in the analysis of ACNs.

## Non-Destructive Techniques and Food Quality Evaluation

With the rapid increase in the global population, the provision of nutrient-dense foods is becoming an emerging challenge (25). Therefore, there is an increased focus on establishing reliable methods for authenticating several quality parameters of agricultural products including internal and external attributes. There are several different conventional techniques available for the assessment of ACNs that are already established such as gas chromatography (GC), high-performance liquid chromatography (HPLC), gas chromatography-mass spectrometry (GC-MS), and liquid chromatography with tandem mass spectrometry (LC-MS/MS). However, these techniques are time-consuming, laborious, require expert knowledge, and complex sample preparation with reagents that can cause environmental pollution and can present a health hazard.

The use of non-destructive spectroscopic techniques, including vibrational methods, possesses a wide range of benefits such as chemical-free assessments, minimal and non-interruptive sample processing, and very rapid and relatively accurate determination of multiple physicochemical properties. Moreover, the advancement in computation, instrumentation, and chemometric methods have further increased the reliance on these rapid assessment techniques in various research domains (26, 27). Some of the spectroscopic techniques include nuclear magnetic resonance spectroscopy (NMR), Fourier transforms infrared spectroscopy (FT-IR), fluorescence spectroscopy (FS), near-infrared spectroscopy (NIRS), hyperspectral imaging (HSI), ultraviolet-visible spectroscopy (UV-Vis), and Raman spectroscopy (RS). The use of these spectroscopic techniques is already widely applied in food (fish, meat, fruit, and vegetable) and beverage (milk, wheat plantlet juice, and tomato juice) analysis (28–32). In addition to spectroscopic techniques, the use of spectral imaging techniques has also been proposed as an option for the quantitative and qualitative analysis of food products (33). Spectral imaging techniques integrate digital imaging and spectroscopy into a powerful analytical system; enabling the acquisition of both spectral and spatial data simultaneously from a target region. Such approaches possess the potential to provide a generic pixel (x, y) and specific wavelength (l), and create a two-dimensional (2D) or three-dimensional (3D) dataset that contains many images of a sample for rapid screening (26, 34). Examples of these imaging techniques are Raman hyperspectral imaging, laser backscattering imaging, microwave imaging, fluorescence imaging, thermal imaging, and odor imaging among others (35–37).

## Chemometric-Based Measurement Through Machine Learning and Artificial Intelligence

**Figure 1:**
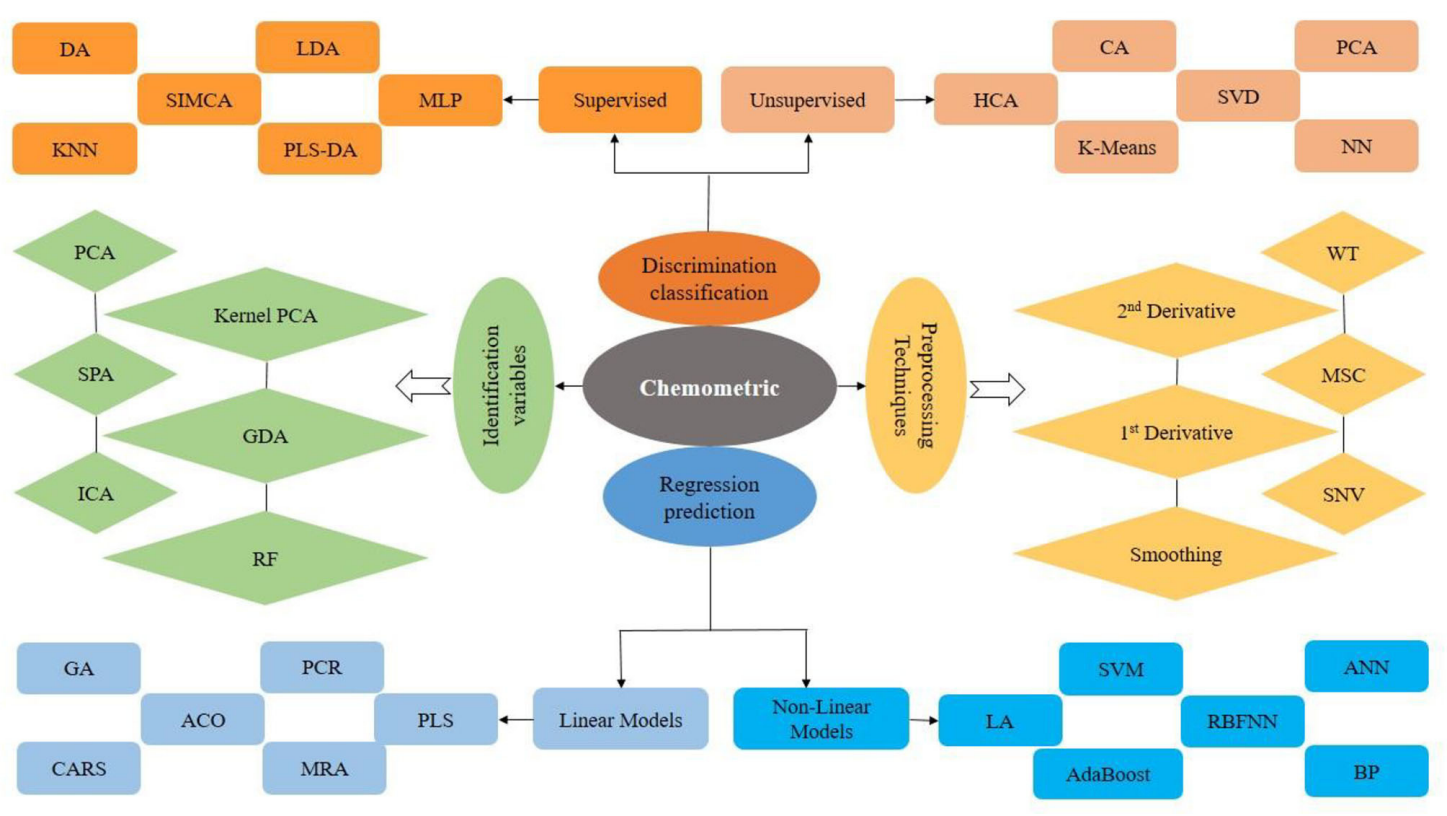
Chemometric models used in spectral imaging and spectroscopic techniques for classification and regression models.

Non-destructive assessment techniques can produce a large amount of information that can effectively be exploited by machine learning, artificial intelligence (AI), and traditional technique-based chemometrics (33). Chemometrics can be described as the science of concerning measurements based on a system or chemical process *via* the use of mathematical or statistical methods. Chemometrics is not a single technique but it is a range of methods that includes signal processing, pattern recognition, curve fitting, statistical analysis, calibration, validation, prediction, preprocessing, and so on. Chemometrics is categorized into quantitative (multivariate calibration) and qualitative analysis (pattern recognition). Pattern recognition is generally utilized for the classification of data analysis and can be supervised or unsupervised as presented in Figure 1 (38). Chemometrics is discussed here as pre-processing technique, classification methods, selection of effective variables from data, and prediction development models (39). AI and machine learning (ML) have combined as the basic technology for qualifying demand-side response. AI has the potential to deal with consumer preferences, challenges, and attributes, ranging from the selection of an optimum set of consumer responses. AI highlighted the expansion of intelligence machines, working and thinking like human beings, for example, planning, learning, problem-solving, and speech recognition. AI is a combination of different phenomena and methods, among which two main concepts known as neural networks and deep learning are supposed to be main tools for better attainment with more advancement (40).

The data obtained from several non-destructive assessment techniques, in general, contain unnecessary information commonly referred to as “noise” that can interfere with the measurement making it inaccurate. Currently, different pre-processing techniques of data have been adopted to create effective models, such as multiplicative scatter correction (MSC), smoothing, standard normal variate transformation (SNV), normalization, orthogonal signal correction (OSC), centering, straight-line subtraction (SLS), wavelet transforms (WT), direct orthogonal signal correction (DOSC), and first and second derivatives (40). The data obtained from each technique are handled based on their characteristics.

Nowadays, there are different types of techniques available for classification, data removal, and regression. The authentication or discrimination with the use of different models plays an important role in the assessment of ACN data collected from conventional and non-destructive techniques. Application of different models for ACNs is generally used such as supervised approaches (*k* nearest neighbors (KNN), soft independent modeling of class analogy (SIMCA), linear discriminant analysis (LDA), multi-Layer Perceptron (MLP), and partial least-squares discriminant analysis (PLS-DA)) and unsupervised approaches (principal component analysis (PCA), hierarchical cluster analysis (HCA), cluster analysis (CA), singular value decomposition (SVD), and neural networks (NN)) (33, 41, 42).

The development of quantitative predictive models was applied for the prediction or quantification of ACNs with non-destructive techniques. Artificial neural network (ANN), backward interval-PLS (Bi-PLS), non-parametric algorithm (NPA), principal component regression (PCR), chaotic neural network (KIII), partial least squares (PLS), competitive adaptive reweighted sampling (CARS)-PLS, response surface regression (RSR), multiple linear regression (MLR), back propagation neural network (BPNN), step multiple linear regression (SMLR), ant colony optimization (ACO)-PLS, genetic algorithm-PLS (GAPLS), and synergy interval-PLS (Si-PLS) (Figure 1) are generally used for quantification models (43–47).

The evaluation of a final build model is essential to judge the predicted effectiveness, accuracy, and reliability for the practical application of the model. In this case, the calibration set is normally used for training, while the prediction set is commonly employed to evaluate the stability and robustness of the model/system. The lowest root mean square error of cross-validation (RMSECV) and root mean square error of the prediction (RMSEP) are frequently adopted to assess the build model performance. The best model can be judged by the lowest RMSEP and RMSECV, or sometimes RSMEC while higher correlation coefficient of calibration and prediction correlation coefficient of calibration (R_C_) and correlation coefficient of prediction (Rp) (48). The model is considered more robust and better if the difference between R_C_ and Rp or RMSECV and RMSEP is small. Furthermore, residual predicted deviation (RPD) shows the build model stability. The stability of the build model can be assessed if RPD <2 (poor), RPD >2 (good), RPD >3 (stable), and RPD >10 (excellent) (49).

## Rapid Non-Destructive Techniques and ACNs Assessment

Several different agricultural products have very similar antioxidant attributes due to the occurrence of bioactive compounds that can potentially prevent cellular oxidation such as carotenoids, flavanols, and stilbenes. However, these functional components are unstable on exposure to physicochemical challenges during the extraction and require specialized analysis commonly using different chromatographic techniques. Therefore, to ensure quality assessment of bioactive compounds, non-destructive spectroscopic and imaging methods have been seen as the best available options, in particular, for their ease of use and non-destructive nature of the techniques deployed (Tables 1, 2) in different food and agricultural products.

**Table 1:** FTIR, NIR, HIS, and RS techniques for the measurement of anthocyanin.

**Technique**	**Food item**	**Attribute**	**Pre-processing and mathematical model**	**Results**	**References**
FT-IR	Red grapes	Total anthocyanins	ANOVA, *Post-hoc* analysis, PCA	Best calibration statistic obtained for merlot grapes SEP = 0.12mg/g and *R*^2^ = 0.84	(50)
	Soybean seed	Anthocyanins	PLSR, MSC, and SNV	*R^2^* of 0.86-0.88, SEP of 9.7-21.8%	(51)
	Soybean seed	Anthocyanins	PLSR, MSC, and SNV	*R^2^* of 0.88-0.90, SEP of 9.4-19.5%	
	Red wine	Anthocyanins	MPLSR, and PCA	*R^2^* for both cross-validation and calibration exceeding 0.8	(52)
	Wine	Anthocyanins	PLSR, and PCA	*R*^2^ = 0.64-0.93, %CVE 25-30%, SEC (mg/L) 0.2-25.5	(53)
	Red grape musts	Anthocyanins	PLSR, and PCA	*R*^2^ = 0.46-0.66, SEC (mg/L) 0.12-16.40, %CVE 15.4-35.4	(54)
NIR	Elderberry	Total anthocyanin	PLSR model for evaluation	RMSECV/RMSEC of 1.31 and RSD_PLSR_ of 13.5%, for pH-differentiation; RMSECV/RMSEC of 1.28 and RSD_PLSR_ of 12.9% for the HPLC	(55)
	Blueberry	Anthocyanins	PLSR, MSC, PCA	RMSECV = 0.25 mg malviding/g and RMSEP = 0.22 mg catechin/g	(56)
	Intact fruit (açaí and palmitero-juçara)	Total anthocyanin	PLS, iPLS, GA, SPA	RMSECV 13.8 g/kg, RMSEP 4.8 g/kg, and *R*^2^ = 0.90	(57)
	Flowering tea	Total anthocyanin	ACO-iPLS, GA-iPLS	*R* = 0.9856, RMSECV = 0.1198 mg/g	(58)
RS	Plum	Anthocyanin and other bioactive compounds	PLSR, SVM	FT (200-1800 wavenumber/cm^−1^), NIR (900-1700 wavenumber/nm), and MIR (800-1800 wavenumber/cm^−1^) SVM-anthocyanin training, validation, and test set accuracy 100%; RMSECV of 9.8 mg/100g, RMSEC of 6.7 mg/100g, and R^2^ *=* 0.9,856	(59)
	Blueberry	Anthocyanin	–	FT-Raman spectra (400-4,000 cm^−1^); n_2_=-2.07 X 10^−7^ cm^2^/W, β=-2.19 X 10^−3^ cm/W, and OL behavior 3.21 X 10^2^ W/cm^2^	(60)
	Purple yam	Anthocyanin	Pearson correlation, multiple dimensional scaling, and hierarchical clustering	Raman spectra (1–3,500 cm^−1^); Identify the major anthocyanin components as cyanidin derivative	(37)
	Bulgarian red wine	Anthocyanin, phenolic, and flavonoid	PCA, PLSR	Raman spectra (400–3000 cm^−1^); 6.6 to 466.8 mg/dm^3^ anthocyanins	(61)
	Blueberries	Anthocyanin	PCA, KNN, PLSR	Raman spectra (900–1800 cm^−1^); R^2^ cal = 0.78/0.87, RMSEC = 43/7.7 mg/hg, R^2^ val = 0.75/0.84, RMSECV = 48/8 mg/hg	(62)
HSI	Purple-fleshed sweet potato	Anthocyanin	LS-SVM, PLSR, MLR	MLR yielded better results; coefficient of determination for prediction ($R_{\text{P}}^{2}$) and calibration ($R_{\text{C}}^{2}$) of 0.866 and 0.868, respectively.	(35)
	Lychee pericarp	Anthocyanin	SRA, SPA, SWR, RBF	RMSEs of 0.610% and 0.567%, and higher coefficients of determination (R^2^) 0.872 and 0.891	(63)
	Strawberry	Anthocyanin	MLR, BNN, RF, NB, SVM	*R^2^* = 0.65	(64)
	Dry black goji berries	Anthocyanin	PLS, LS-SVM, PCA, WA, CNN	For PLS: $R_{\text{C}}^{2}$ = 0.914, RMSEC = 103.03, $R_{\text{v}}^{2}$ = 0.916, RMSEV = 104.21, $R_{\text{p}}^{2}$ = 0.883, RMSEP = 123.89 For LS-SVM: $R_{\text{C}}^{2}$ = 0.934, RMSEC = 89.813, $R_{\text{v}}^{2}$ = 0.893, RMSEV = 120.786, $R_{\text{p}}^{2}$ = 0.875, RMSEP = 130.48 For CNN: $R_{\text{C}}^{2}$ = 0.929, RMSEC = 97.29, $R_{\text{v}}^{2}$ = 0.931, RMSEV = 92.48, $R_{\text{p}}^{2}$ = 0.889, RMSEP = 118.24	(65)
	Wine grapes	Anthocyanin	PLSR, SVR	For SVR: $R_{\text{p}}^{2}$ = 0.9,414, RMSEP = 0.0046 For PLSR: $R_{\text{p}}^{2}$ = 0.8,407, RMSEP = 0.0129	(66)
	Grape berry	Anthocyanin	ε-SVMs	$R_{\text{p}}^{2}$ = 0.83, RMSE of 0.211 mg/g berry, $R_{\text{v}}^{2}$ = 0.72, RMSE of 0.282 mg/g berry	(67)

*PLSR, partial least squares regression; SEC, standard Error of Calibration; CVE, errors of cross validation; SPA, successive projection algorithm; SVR, support vector regression; MSC, multiple scatter correction; SNV, standard normal variate; SWR, stepwise regression; RBF-SVR, radial basis function support vector regression; RBF-NN, radial basis function neural network; SWR-RBF-SVR, stepwise regression-radial basis function-support vector regression; SPA-RBF-SVR, successive projection algorithm-radial basis function-support vector regression; GA, genetic algorithm; RMSEs, root mean square errors; RMSEP, root mean square error of prediction; RMSEV, root mean square error of validation; PLS, partial least squares; LS-SVM, least-squares support vector machine; CARS, competitive adaptive reweighted sampling, n_2_, nonlinear index of refraction; β, nonlinear absorption coefficient; OL, optical limiting; KNN, K-nearest neighbor; BNN, back propagation neural network; RF, random forest; NB, naive Bayes; SVM, support vector machine; WT, wavelet transform; CNN, convolutional neural networks; ε-SVMs, epsilon-support vector machines; and PCA, principal component analysis*.

**Table 2:** NMR, FS, RI, and UV-Vis techniques for the measurement of anthocyanin.

**Technique**	**Type**	**Sample**	**Attribute**	**Purpose**	**Reference**
NMR	Spectroscopic	Grape berry skin	Anthocyanin	identification and composition	(68)
	Spectroscopic	Red wine	Anthocyanin-flavanol	Identification	(69)
	Spectroscopic	Boysenberry	Anthocyanin	Structural identification	(70)
	Spectroscopic	Purple corn varieties	Flavanol-anthocyanin	Characterization and presence	(71)
	Spectroscopic	Purple sweet potato	Acylated anthocyanins	Isolation and identification	(72)
	Spectroscopic	Black seed coated Korean adzuki bean	Anthocyanin	Identification and composition	(73)
	Spectroscopic	Aging red wine	Acylated anthocyanin-vinyl-flavanol	Structural characterization	(74)
	Spectroscopic	Aged red wines	Anthocyanin-derived pigments	Isolation and characterization	(75)
FS	Spectroscopic	purple corn	Anthocyanin	Impact of alginate and zinc ion on the chemical stability of anthocyanins	(76)
	Spectroscopic	Black Soybean	Anthocyanin	Chelating Activity of Anthocyanin	(77)
RI	Imaging	Bilberry, elderberry	Anthocyanidins	Identification of anthocyanins without glycosidic moiety	(78)
	Imaging	Bilberry, elderberry, sumac, purple corn, and hollyhock	Anthocyanin	Identification of anthocyanins	(79)
UV-Vis	Spectroscopic	Graphs	malvidin-3,5-O-diglucoside	Extracted anthocyanins from samples and tested for antioxidant potential	(80)

### Nuclear Magnetic Resonance Spectroscopy

The implementation of NMR-based methods (low and high-filed NMR) has received increased attention and consideration in the food industry (81). NMR is a relatively robust technology and has been commonly used for the authenticity and traceability of several different food samples without the modification of the food sample and generation of wastes (82). This approach provides the opportunity to estimate the structural data quantitatively, by carrying atoms with an internal angular momentum and magnetic moment. Moreover, low-field proton (^1^H-NMR) relaxation is continuously utilized to quantify and distinguish a large number of compounds at the same time (83). However, the challenge of several agricultural food products is that in addition to being chemically and structurally heterogeneous structures, every compound can have distinctive participation in the NMR signal principally due to variations in molecular movement (83).

For the past few decades, NMR spectroscopy has been used in the food industry for structural characteristics, compositional analysis of functional components, food authentication, physicochemical analysis, and microbiological inspection of various food and agricultural samples (72). The use of NMR can also be applied for ACN detection in food items. A study by González-Manzano et al. (71) isolated the anthocyanin-flavanol condensed pigment from purple corn powder, and later its structure was examined by ^13^C and ^1^H NMR using correlation spectroscopy, heteronuclear multiple bond correlation, and heteronuclear single quantum coherence techniques. The existence of condensed pigments was observed in three out of six corn varieties and the condensed pigment results ranged from 0.3 to 3.2% of total ACNs. A study by Zhang et al. (72) established an efficient approach for the scale formation of high-purity ACNs mixtures and a semi-preparative HPLC method was used to obtain monomeric acylated ACNs. This was followed by the use of ^1^H and ^13^C-NMR to allow the specific connectivity of acyl and glycosyl moieties and ascertained the entire structure of acylated monomeric ACNs. The specific structures of the unique monomeric acylated ACNs from purple sweet potato, namely peonidin, were determined by ^13^C and ^1^H-NMR.

Similarly, Zielińska et al. (84) determined the variations in ACNs contents and chlorogenic acids during *Aronia* fruit ripening and development. Comparative analysis using HPLC-DAD and NMR spectroscopy was performed for the determination of chemical composition and data were examined using multivariate statistics and chemometric analysis. The ^1^H-NMR spectrum revealed different signals in the aromatic protons varying between 6.5 and 9.0 ppm proposed to represent the ACNs content, which begins to appear in the second stage of fruit development along with a dark color. For ripe fruit extract, a two-dimensional (^1^H-^1^H COZY) NMR spectrum presented H5'/H6', H2'/H6', and H4/H8 correlations, and the resonances of two major ACNs, cyanidin arabinosides, and cyanidin galactosides were also reported. Relatively recently, a study by Ha et al. (73) also investigated the ACNs derivatives (delphinidin-3-*O*-glucoside and delphinidin-3-*O*-galactoside) in the black seed coated adzuki bean through NMR spectroscopy.

### Fourier Transform Infrared Spectroscopy

One of the most extensively used methods for real-time, reliable, and rapid evaluation of food products without requirements for extensive training and operating skills is FT-IR. The FT-IR analysis is based on the principle that functional groups within the phytochemicals require different energy levels for excitation (20). In the IR region, the electromagnetic spectra can be broadly classified into near-infrared (NIR) spectra (4,000 to 12,821 cm^−1^), mid-infrared (MIR) (400 to 4000 cm^−1^), and far-infrared (FIR) spectra (33 to 400 cm^−1^) (85). By obtaining data about chemical components and producing authentic fingerprints, the high-energy FT-MIR and FT-NIR spectra are proposed to be more fitting as compared to FT-FIR spectra for inspecting the quality of food products (86). It is proposed that the FT-MIR spectra reveal the information about molecular fundamental vibrations and associated rotational vibrational structures, whereas the FT-NIR spectra provide more complex structural information because they can excite overtone or harmonic vibration as the vibrational behavior of bond combinations (87). However, low-moisture products, such as cereals, possibly need dilution in any medium due to comparatively high absorption coefficients presented by various elements in the MIR region, which restricts the FT-IR industrial applications (20). The FT-IR analysis of vegetables and fruits can be classified into two groups requiring analysis: raw food products and/or processed materials such as extracts, juices, and purees (88). In addition, the use of FT-IR techniques can be successfully implemented in the food industry for the analysis of different substances in the evaluation of food forensics and potential adulteration of different food products (89). The use of FT-IR is also suitable for the detection of ACN content in different agricultural samples. A study by Miramont et al. (52) examined the concentrations of different ACNs by FT-IR spectroscopy using PLS models during red wine fermentation. The observed results have indicated that the determination coefficient (*R*^2^) for both cross-validation and calibration exceeds 0.8. Furthermore, the authors have also proposed that FT-IR spectroscopy with PLS regression used in this estimation of various ACNs parameters is a reliable and adequate technique for providing winemakers with indications for keeping the suitable conditions that can affect wine quality and pigment concentration.

A study by Amanah et al. (51) evaluated the feasibility of FT-IR and FT-NIR to estimate different types and total ACNs content in soybean seeds. The spectra were obtained from 70 distinct types of soybean seeds and data were correlated with the chemical components analyzed using HPLC-DAD. Several pre-processing methods including data normalization, MSC, first and second derivatives, and SNV were used to develop an optimal PSL regression model to predict the ACNs content. The prediction performance of the PLS regression models for FT-IR spectra showed *R*^2^ = 0.86–0.88 and standard error of prediction (SEP) (9.7–21%) for chemical components, which were lower than FT-NIR delivering, *R*^2^ = 0.88–0.90 and SEP (9.4–19.5%). The results of this study indicated the relatively strong and reliable potential of FT-IR and FT-NIR techniques to non-destructively predict ACNs contents in a single seed of soybean (51).

### Fluorescence Spectroscopy

**Figure 2:**
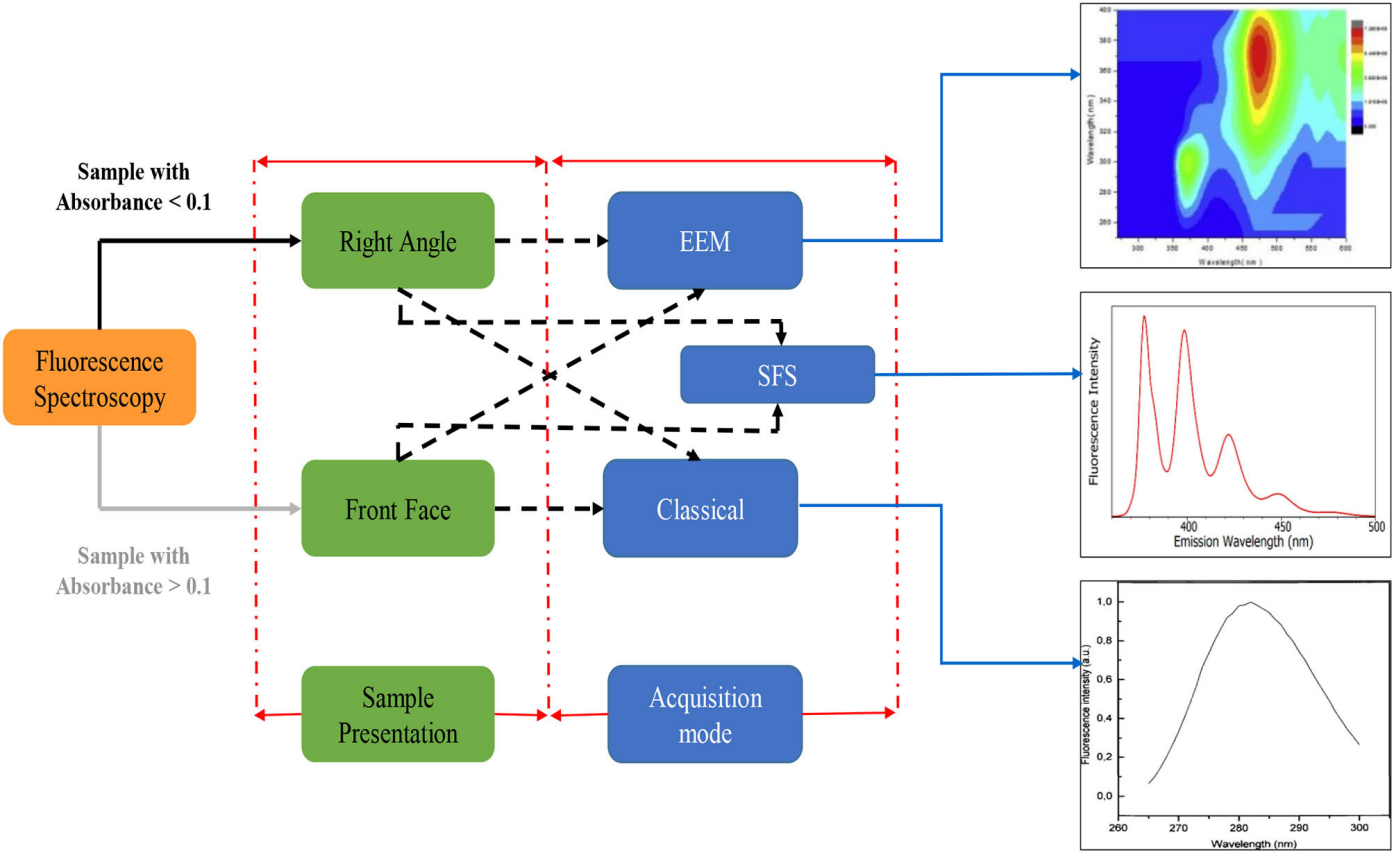
Fluorescence spectroscopy principal acquisition mode; EEM, Excitation emission matrix; SFS, Synchronous fluorescence spectroscopy.

Fluorescence spectroscopy is a sensitive and rapid assessment technique used to evaluate the various food components based on a particular fluorescence generated from the fluorophore materials. A fluorophore is an organic molecule that can absorb light at a distinct wavelength and then release it at a higher wavelength. Different fluorophores emit particular excitation and emission peaks that can be ascribed to different compositional and structural variations in a sample (90). In the food industry, FS is commonly used to determine lipid oxidation, Maillard products, vitamin A, and chlorophyll (91–93). The fundamental concept of FS is presented in Figure 2. Moreover, the technique is widely used to determine the structural changes in proteins when interacting with different phytochemicals such as ACNs. For example, a study by Dumitrascu et al. (94) investigated the interaction of heated soy proteins with ACNs from cherry fruit using FS. Furthermore, an *in silico* method was also used to highlight the interaction between ACNs and soy proteins. The docking outcomes further supported the FS findings showing affinity to glycinin for cyanidin 3-glucoside and cyanidin 3-rutinoside (94).

A study by Condurache et al. (95) investigated the ACNs composition of eggplant peels extract for binding properties with bovine peptides using the FS quenching approach and molecular modeling method. The *in-silico* method was performed to determine the single-molecule level interaction between various peptides and main ACNs. The results showed fluctuation in fluorescent attributes of lactoferrin hydrolysate with the addition of ACNs.

### Near-Infrared Spectroscopy

**Figure 3:**
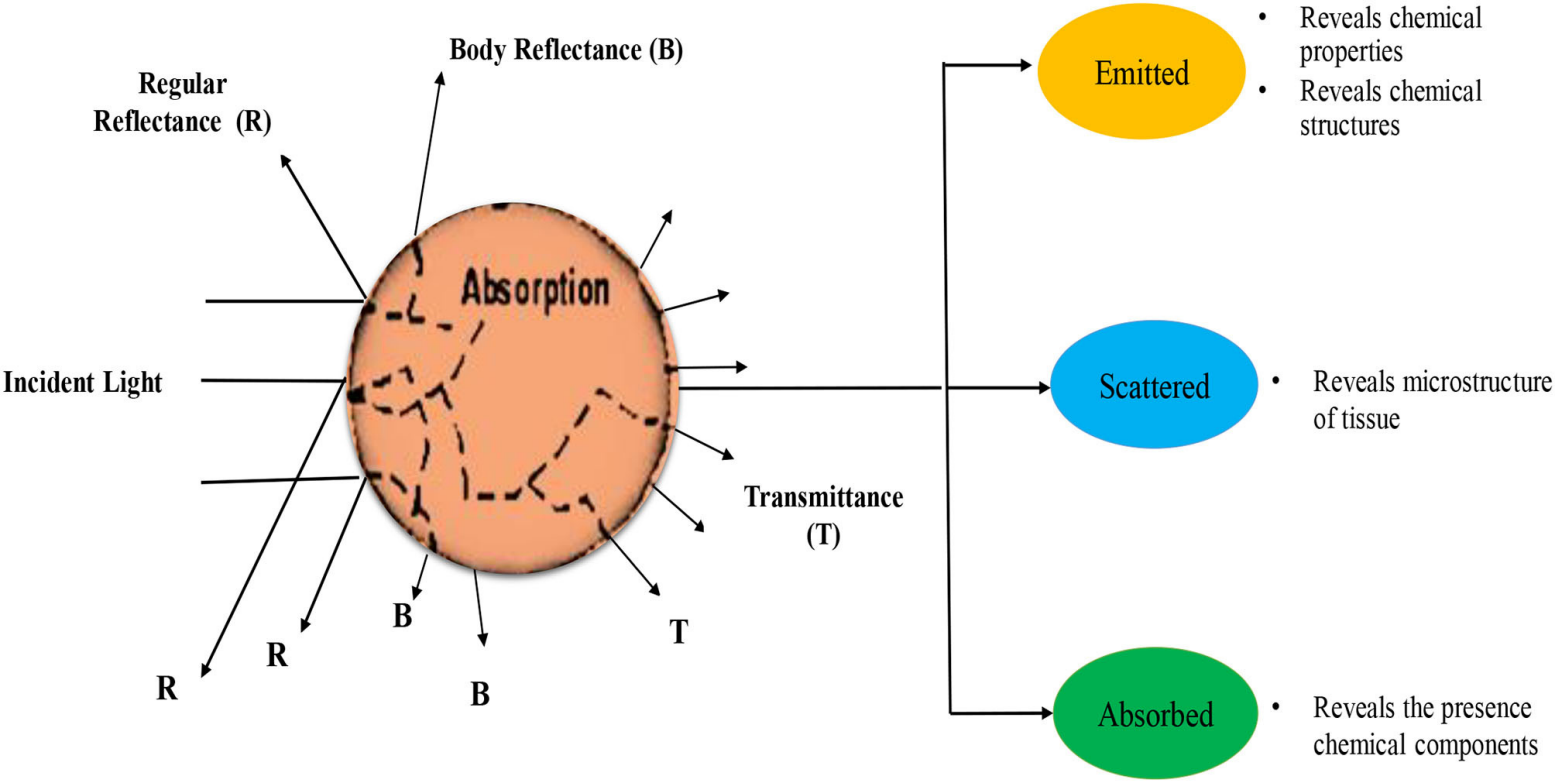
A fundamental concept of near-infrared spectroscopy.

The NIRS is a rapid analytical technique that utilizes analytes absorption mechanism (radiations) of the spectra in the range between 700 and 2500 nm (96, 97). This analytical approach (Figure 3) ascertains that the C-H, N-H, and O-H vibrational motions correspond with the chemical composition of a sample (98, 99). Furthermore, the NIR radiation can interrelate with samples by interacting, reflectance, or transmittance modes. The interacting mode describes the mechanism which estimates the materials' responses at a laterally different point on the surface of a sample while the transmittance mode evaluates the NIR spectra that proceed through the sample. In contrast, the reflectance mode of NIR evaluates the bands that are reflected from the material (100). The NIRS is reported to be suitable for the assessment of ACNs in food and agricultural products. A study by Stuppner et al. (55) used the NIRS method for the determination of total ACNs in whole elderberry fruits. The results were also compared with the pH-differential method and ultra-high performance liquid chromatography-multiple wavelength detection-ultra high resolution-quadrupole-time of flight-mass spectrometry (UHPLC-MWD-UHR-Q-TOF-MS). In this study, pre-processing methods included data smoothing (second derivatives), and SNV was used to develop an optimal PLS regression model to predict the ACNs content. The findings have indicated that relative standard deviation (RSD_PLSR_) and root mean square error of calibration (RMSECV/RMSEC) for NIRS-calibrated pH-differentiation method was 13.5% and 1.31 and for uHPLC 12.9% and 1.28, respectively. Furthermore, the NIRS estimation appeared to be a cost-efficient and more reliable technique for the measurement of ACNs in whole elderberries. Likewise, a study by Inácio et al. (57) used NIR reflectance spectroscopy to evaluate ACNs in palmitero-jucara and intact acaí fruit. Different preprocessing methods (Savitzky-Golay smoothing, first polynomial order, mean-centering, and first and second derivatives), chemometric regression models (PLS-iPLS, Full-PLS, MLR-SPA, PLS-SPA, and PLS-GA), variable selection methods (SPA, GA, and iPLS), and system validation were performed to develop an optimal PLS regression model to predict the ACN content. The obtained results also indicated the PLS regression model, as accuracy measured as RMSEC=13.8, RMSEP=4.8, $R_{p}^{2}$= 0.90, $R_{c}^{2}$= 0.97, and RPD=3.08. These results propose that NIRS and multivariate calibration are effectively employed to determine ACN content in palmitero-juçara and intact açaí fruit (57).

A study by Xiaowei et al. (58) estimated total ACNs using the NIR method with a spectrum ranging from 4000 to 10,000 cm^−1^. Genetic algorithm interval PLS and ant colony optimization interval PLS were applied to develop calibration models for ACNs. The ant colony optimization interval PLS model for total ACNs (RMSECV=0.1,197 mg/g and *R*^2^ = 0.9,857) had better results than genetic algorithm interval PLS, full-spectrum PLS, and iPLS models. These outcomes propose that NIRS can be employed for the non-destructive estimation of total ACNs content.

The major disadvantage of NIR spectroscopy is the interference with the samples that contain high water content. These samples may generate a strong absorption band in a definite spectral range which limits its potential commercial application (101). Although this method may be inappropriate for the measurements of volatile components in fruits, it is still integrated with an “electronic nose” for the detection of volatile components. Therefore, this type of approach depends on the standardized size of the sample, homogeneity, temperature, and presentation of the analyzed sample (102).

### UV-Vis Spectroscopy

The ultraviolet-visible (UV-Vis) spectroscopy is fast absorption spectroscopy that measures the interaction of light with a sample in the UV and visible spectra between 200 and 780 nm (103, 104). The light can interact with samples by absorption, refraction, diffraction, reflection, or scattering which depends on the functional groups in the materials to be analyzed (105). This technique can be used for the identification and relatively accurate quantification of different constituents in food samples (106, 107). This method is also suitable for the detection of coloring compounds such as ACNs profiling. A study by Nistor et al. (108) extracted ACNs from chokeberries and black carrots using different solvents and characterized them with UV-Vis coupled to HPLC integrated with MS. From chokeberries, five bioactive monoglycosylated ACNs were determined, while four acylated and four diglycosylated compounds were identified from black carrot extracts. In a study by He et al. (80), various ACNs from *Vitis amurensis Rupr* (a graph variety) were extracted and identified by UV-vis spectroscopy, FTIR spectroscopy, MS, and NMR.

### Hyperspectral Imaging

Hyperspectral imaging (HIS) is a fast analytical technique that integrates optical spectroscopy and digital imaging principles with spectral and spatial information simultaneously from a material in a 3D dataset (109, 110). Although HSI is the extension of multispectral imaging (MSI), it is relatively slower than multispectral imaging as it includes extended scanning time for online analysis and evaluation (111). The images from HSI can be obtained by area, line, and point scanning methods. In area scanning, simultaneously, a 2D single-band grayscale image with full information is obtained. In the point scanning method, spectral information is scanned, while the inline scanning method incorporates the slit of an area with complete spectral information and is an extension of the point scanning method (112). This imaging method has also been found to be suitable for ACN detection in various samples. A study by Yang et al. (63) proposed a quantification approach for the estimation of lychee pericarp ACNs using HSI. Sense-Reason-Act (SRA) and successive projection algorithms (SPA) were used to overcome data dimensionality, while for a 3-D lychee image, an HSI system in the range of 350 to 1,050 nm was employed. To enhance the prediction accuracy of stepwise regression-radial basis function-support vector regression (SWR-RBF-SVR), successive projection algorithm-radial basis function-support vector regression (SPA-RBF-SVR) models were fused into a single model by the radial basis function neural network (RBF-NN) algorithm. The fused model exhibited lower RMSEs of 0.610% and 0.567% and higher coefficients of determination (*R*^2^) of 0.872 and 0.891 for the testing and training sets, respectively (63). This research supported the notion that the HSI method is proficient in visualizing and predicting the ACNs contents in lychees.

The use of HSI for ACNs detection in purple-fleshed sweet potatoes was evaluated in a study by Liu et al. (35). The PLSR and LS-SVM were used to build the calibration models based on raw spectrum and spectrum preprocessed by Savitzky–Golay filter, moving average, SNV, and MSC methods. Three different algorithms such as LS-SVM, PLSR, and multiple linear regression (MLR) were used to establish models based on 10 optimal wavelengths adopted by the regression coefficients (RC) method. The obtained results proposed that RC-MLR yielded better results with $R_{P}^{2}$ and $R_{C}^{2}$ of 0.866 and 0.868, respectively.

A study by Zhang et al. (65) measured total ACN content in black goji berries by near-infrared HSI technique. The CNN model was built to estimate the chemical compositions of black goji berries. The wavelength selection methods (SPA and CARS), modeling methods (LS-SVM and PLS), and extraction methods (wavelet transform and PCA) were studied as conventional methods for comparison. For total ACNs, the performance of RPDp and RPDv of all models were over 2.000 and *R*^2^*p, R*^2^*v*, and *R*^2^*c* of all models were over 0.850. In this study, deep learning and feature extraction methods provided comparable results with the conventional data analysis methods (65).

Furthermore, a study by Chen et al. (66) quantified ACNs values in wine grapes during the ripening stages using the HSI. The spectral images were recorded with a range of 900 to 1,700 nm and a quantitative model for ACNs determination was developed using the SVR or PLSR. The SVR model showed better results than the PLSR model, as it yielded RMSEP of 0.0,046, and a coefficient of validation [P*-*R (2)] of 0.9,414 much higher than the PLSR model, which yielded RMSEP of 0.0,129 and [P*-*R (2)] of 0.8,407.

### Raman Spectroscopy

Raman spectroscopy (RS) is a label-free vibrational spectroscopic method that provides information about the chemical bond, molecular structure, and molecular composition of the analyte. This type of analysis was widely used in pharmaceutical and medical sciences, detection of bioactive compounds, molecular composition, and several other diagnostic purposes (113). The RS system is composed of a narrow-band laser, data controlling software, data processor, and electrical and optical components. The laser used in this spectroscopy causes the fluorescence of organic compounds, influencing the signal-to-noise ratio and reducing the sensitivity of the technique. Usually, the electromagnetic radiations emitted are collected with the lens and passed through the collimator. The molecules from the ground move to an excited state and then into a vibrational state usually referred to as anti-stoke Raman scatter. A change in the polarity of molecules is required for Raman scattering (19).

Relatively recently, a study by McIntyre et al. (59) used different spectroscopic techniques (Raman, mid-infrared, and near-infrared) in combination to assess the range of bioactive compounds (including ACNs) in different plum cultivars. The obtained spectra were first pre-processed through SVN, second-order polynomial, Savitzkyp–Golay, and 5-point window to remove differences associated with spectral intensity. Then, PLSR was used to compare the data obtained from spectroscopic techniques and conventional methods. PLSR of Raman data indicated the best results for ACNs with a test set RMSEP of 12 mg/100 g than the one obtained with MIR and NIR.

Furthermore, a study by Jeyaram and Geethakrishnan (60) identified the functional group of ACNs using FT-Raman FT-IR. Results showed that ACNs contents present a negative non-linear index of refraction (n_2_) and nonlinear absorption coefficient (β), respectively, due to its saturable and self-defocusing absorption nature. The β and n_2_ values of ACNs were measured to be −2.19 × 10^−3^ cm/W and−2.07 × 10^−7^ cm^2^/W, respectively.

### Surface-Enhanced Raman Spectroscopy

To provide additional strengths to the Raman scattering phenomenon in normal Raman spectroscopy, different nanoparticles were used to boost the Raman peaks and the process is named SERS (100). Several nano substrates are commonly used (gold, silver, and copper) in both food and non-food applications (114). The main reasoning for the use of nanoparticles is that placement of this material near nanoparticles, chemical, and physical mechanisms contribute (simultaneously) to enhance the Raman signals from up to 10^12^ (115). The chemical process occurs due to charge transfer between nanomaterial and sample, while amplification of signals due to optical properties of nanoparticles is a physical mechanism (116). Furthermore, the sensitivity of SERS relies on the nano substrates proposed, and recently hundreds of nanoparticles have been proposed for different food safety investigations and others (115, 117). However, nanoparticles with uniform and stable hot spot region (small area with higher enhancement of signals) remains a challenge. Numerous functionalized nanoparticles have been proposed recently with better stability and sensitivity such as thioglycolic acid-activated nano-substrates (118) and octane thiol-fabricated nanoparticles (119) among others. The functionalized nanoparticles provided more adsorption sites for target samples, which enhanced the target results.

A study by Zaffino et al. (120) proposed the use of different pH ranges (3–10) for the identification of six different anthocyanidins using silver as an SERS substrate. The study proposed that the interaction taking place through the hydroxyl group is due to the aromatic system and quinoidal bases (120). Similarly, silver-based SERS was also used for the determination of anthocyanin from textile dyed and plant sources (purple corn, sumac, elderberry, hollyhock, and bilberry). Four characteristic peaks were observed at 1,330, 1,530, 1,590, and 1,640 cm^−1^ assigned to ring stretching vibrational modes, while a strong peak at ~1,240 was ascribed to the C-OH bond to be from protentional bioactive compounds (79).

## Future Perspectives

**Figure 4:**
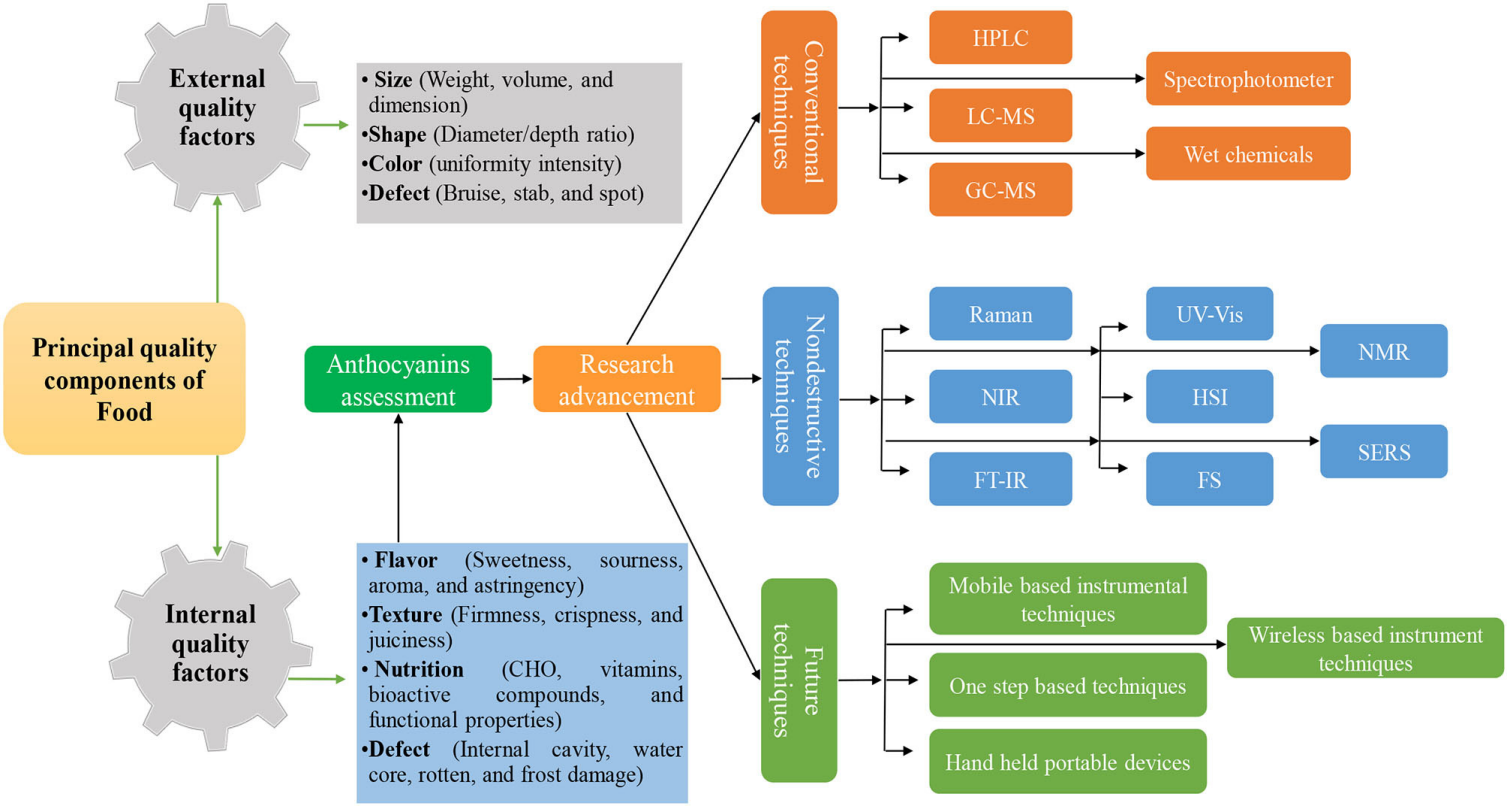
Principal quality components of food and future advancement in non-destructive techniques with time.

Recent research established that rapid spectroscopic and imaging methods have the potential to replace conventional chromatographic and other troublesome methods for detection applications in food samples. However, future work can be focused on minimizing the disadvantages existing in these methods. For example, the synthesis of nanoparticles with more hotspot regions, stability, and cost-effectiveness can be extended to the commercial application of the SERS method. Likewise, the introduction of innovative algorithms to extract target peaks from the redundant data (non-target peaks) in the HSI approach. Similarly, efforts can be also diverted toward less involvement of chemicals used as a reference for calibration of the NIRS equipment in which 20 to 50 chemicals of known values are used. In addition, the integration of two or more methods can also improve the reliability of work. Moreover, coupling spectroscopic and imaging tools with non-thermal methods is also an available option in future studies. All imaging and spectroscopic techniques can have unnecessary information and background elements in spectral data which can influence the result of the expected study. Therefore, a suitable pre-processing method and proper model selection will generate more reliable findings. Moreover, the building of single-step fast algorithms is essential to provide a suitable solution for food safety and quality monitoring. The future researcher should be orientated toward the careful planning and execution of non-destructive techniques for combining the non-invasive nature for the forecast of internal and external food quality parameters (Figure 4). Moreover, the use and generation of portable, easy to handle, low-cost, easy-to-use techniques, and the use of simple and novel chemometric algorithms for data interpretation are essential (Figure 4).

## Conclusion

With the increasing demand for food production and supply to meet the needs of the global population growth, there is an increasing demand for rapid assessments of food quality and safety. Therefore, non-invasive quality assessment technologies relating to the analysis of individual phytochemicals are rapidly developing. In the present review, we have summarized the latest literature relating to the ACNs screening through non-destructive spectroscopic and imaging techniques. The importance of rapid screening methods, their principles, application outlines, and the benefits and some pitfalls of these approaches are also reviewed. Furthermore, the use of chemometrics base AI for possible practical utilization in food and agricultural food products analysis represents one of the potentially greatest advancements in the applications of non-destructive techniques.

## Author Contributions

MM, AH, and NN: writing original article. MR, NA, and EK: reviewing and editing. BX and SI: supervision. All authors contributed to the article and approved the submitted version.

## Funding

This research was funded in part by Grants (project numbers NC.X337-5-21-170-1 and NC.X341-5-21-170-1) from the National Institute of Food and Agriculture (NIFA) and Jiangsu Postdoctoral Fund (2020Z084).

## Conflict of Interest

The authors declare that the research was conducted in the absence of any commercial or financial relationships that could be construed as a potential conflict of interest.

## Publisher's Note

All claims expressed in this article are solely those of the authors and do not necessarily represent those of their affiliated organizations, or those of the publisher, the editors and the reviewers. Any product that may be evaluated in this article, or claim that may be made by its manufacturer, is not guaranteed or endorsed by the publisher.
